# Bone Marrow Osteoblastic Niche: A New Model to Study Physiological Regulation of Megakaryopoiesis

**DOI:** 10.1371/journal.pone.0008359

**Published:** 2009-12-21

**Authors:** Isabella Pallotta, Michael Lovett, William Rice, David L. Kaplan, Alessandra Balduini

**Affiliations:** 1 Department of Biomedical Engineering, Tufts University, Medford, Massachusetts, United States of America; 2 Department of Biochemistry, University of Pavia, Pavia, Italy; Katholieke Universiteit Leuven, Belgium

## Abstract

**Background:**

The mechanism by which megakaryocytes (Mks) proliferate, differentiate, and release platelets into circulation are not well understood. Growing evidence indicates that a complex regulatory mechanism, involving cellular interactions, composition of the extracellular matrix and physical parameters such as oxygen tension, may contribute to the quiescent or permissive microenvironment related to Mk differentiation and maturation within the bone marrow.

**Methodology/Principal Findings:**

Differentiating human mesenchymal stem cells (hMSCs) into osteoblasts (hOSTs), we established an *in vitro* model for the osteoblastic niche. We demonstrated for the first time that the combination of HSCs, Mks and hypoxia sustain and promote bone formation by increasing type I collagen release from hOSTs and enhancing its fibrillar organization, as revealed by second harmonic generation microscopy. Through co-culture, we demonstrated that direct cell-cell contact modulates Mk maturation and differentiation. In particular we showed that low oxygen tension and direct interaction of hematopoietic stem cells (HSCs) with hOSTs inhibits Mk maturation and proplatelet formation (PPF). This regulatory mechanism was dependent on the fibrillar structure of type I collagen released by hOSTs and on the resulting engagement of the alpha2beta1 integrin. In contrast, normoxic conditions and the direct interaction of HSCs with undifferentiated hMSCs promoted Mk maturation and PPF, through a mechanism involving the VCAM-1 pathway.

**Conclusions/Significance:**

By combining cellular, physical and biochemical parameters, we mimicked an *in vitro* model of the osteoblastic niche that provides a physiological quiescent microenvironment where Mk differentiation and PPF are prevented. These findings serve as an important step in developing suitable *in vitro* systems to use for the study and manipulation of Mk differentiation and maturation in both normal and diseased states.

## Introduction

Hemopoiesis occurs in a complex microenvironment within the bone marrow. Megakaryocytes (Mks) and their progeny, circulating anucleated platelets (plts), are vascular cells involved in many aspects of hemostatic and inflammatory functions, as well as the site for many blood disorders. As observed in mice, Mks release platelets through a complex mechanism that converts the bulk of their cytoplasm into multiple long processes called proplatelets [1], [2]. These protrusions are thin and branch repeatedly. Mk maturation and platelet generation occurs in selected environments within the bone marrow, with proplatelet formation following Mk migration from the osteoblastic to the vascular niche, where newly generated platelets can be released into the bloodstream [3].

A growing body of evidence indicates that the characteristics of the microenvironment surrounding Mks plays an important role in the regulation of platelet production within the bone marrow [4], [5], [6]. The vascular niche is comprised of extracellular matrix (ECM) proteins such as collagen type IV, fibronectin, laminin, fibrinogen [7], [8], and most likely VWF, which allow proplatelet formation. Type I collagen, however, completely suppresses proplatelet formation and is the most abundant extracellular protein of the osteoblastic niche [7], [9]. It is known that mutual regulatory interactions occur between Mks and osteoblasts, with Mks contributing to bone homeostasis and osteoblasts supporting megakaryopoiesis through the release of growth factors [10], [11]. However, it is also known that under normal physiological conditions the osteoblastic niche inhibits Mk maturation and differentiation [12], [13], [14]. Therefore, the dynamic interaction of Mks with the different ECM proteins within the bone marrow seems to orchestrate their maturation in specific sites. In addition, we have shown that engagement of receptors is dependent on both the composition and structural properties of these matrices [6], [15].

Underlying these complex regulatory mechanisms is the development of mechanical forces and the activation of biochemical signaling pathways, which may be modulated by biophysical signaling. Oxygen tension, for example, is a modulator of cell activity within the bone marrow, and an oxygen gradient exists between the osteoblastic and vascular niches [16], with more mature cells migrating towards the higher oxygen vascularized compartment of the bone marrow [17], [18]. On this basis, it is clear that a variety of different features contribute to the formation of a quiescent or permissive microenvironment for hemopoietic stem cell differentiation and maturation within the bone marrow space, necessitating a more complex *in vitro* model. Although the development of *in vitro* culture techniques has greatly improved our knowledge of blood cell production, several aspects of hemopoiesis remain unresolved due to the inability of current *in vitro* models to reproduce the complexity of the bone marrow.

The purpose of this study was to establish an *in vitro* model for Mk function in the bone niche environment. First we formed a model that reproduced and combined most cellular, matrix, and physical components that characterize the physiological osteoblastic niche. Further, by including hemopoietic stem cells (HSCs) in the model, we investigated crosstalk between HSCs and cells comprising the osteoblastic niche. This work represents the first step towards the development of a new 3D bone marrow model for understanding basic cell biology and regulatory mechanisms of platelet formation. The long term goal is to utilize this type of model to elucidate new clinical options to control these cells for disease management.

## Results

### *In vitro* reproduction of osteoblastic niche physiological environment

**Figure 1:**
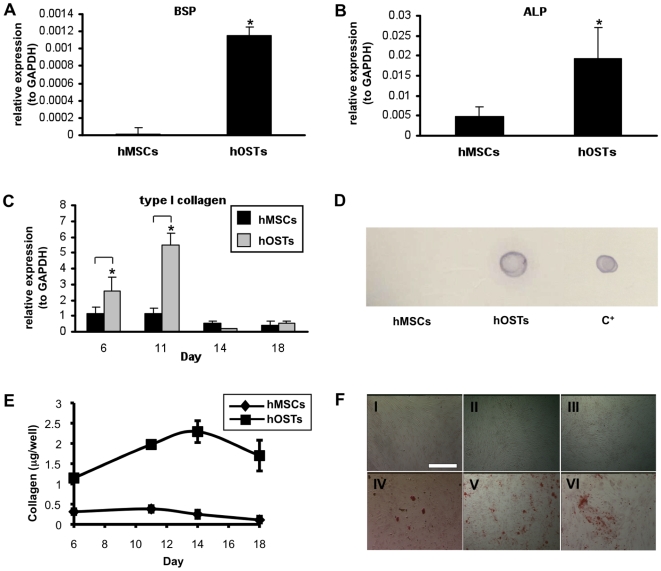
Characterization of the osteoblastic niche. As markers of differentiation we analyzed the expression of bone-related genes, BSP (A), ALP (B) and type I collagen (C) by hOSTs after 6 days of differentiation, as compared to hMSCs, evaluated by real-time RT-PCR. Error bars represent standard deviations. (C) Time course analysis of type I collagen expression by hMSCs and hOSTs at different culture time, evaluated by real-time RT-PCR. (D) Type I collagen protein expression by hMSCs and hOSTs at day 11 of culture. 10 µg of hMSCs and hOSTs lysate and 40 ng of purified type I collagen (C^+^) were employed in a dot-blot assay. (E) Time course analysis of type I collagen release by hMSCs and hOSTs. The amount of hydroxyproline was measured and then multiplied by a factor of 7.46 to give the total collagen for each well, as described in Materials and Methods. (F) Mineralization of hMSCs (I, II, III) and hOSTs (IV, V, VI)) was evaluated by alizarin red staining, after 6, 11 and 18 days of differentiation (scale bar  = 200 µm). The cultures were performed in normoxic conditions, data are the mean ± SD of the results obtained in three different experiments for each sample. *p<0.05.

To develop a physiological osteoblastic niche to study hemopoiesis, human mesenchymal stem cells (hMSCs) were differentiated towards the osteoblastic (hOST) lineage for six days, corresponding to the upregulation of the bone-related genes: bone sialoprotein (BSP), alkaline phosphatase (ALP) and type I collagen (Figure 1A–C). In addition, a time course analysis revealed that type I collagen expression by hOSTs peaked after eleven days of differentiation and rapidly decreased afterwards (Figure 1C). These results were also confirmed at the protein level by dot blot analysis that revealed type I collagen only in hOSTs after 11 days of culture (Figure 1D). Consistently, type I collagen was progressively released by hOSTs with a peak after fourteen days of culture, as revealed by hydroxyproline assay (Figure 1E). Finally, in Figure 1F we show that only hOSTs, and not hMSCs, presented calcium depositions over time.

**Figure 2:**
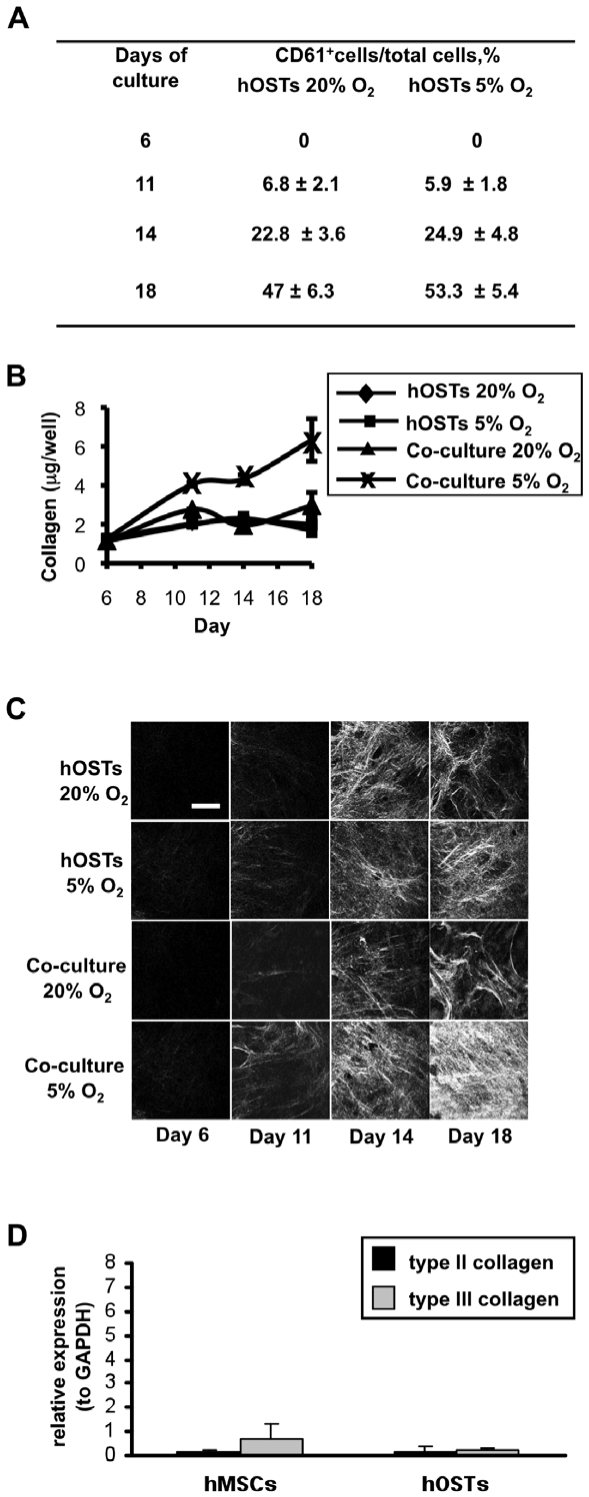
Contribution of HSCs, Mks and hypoxia on type I collagen release and organization. (A) HSC differentiation into Mks was monitored over time and % of CD61^+^ are reported for each time point. (B) Collagen produced by hOSTs alone or in direct co-culture with HSCs, at different oxygen tensions. The amount of hydroxyproline for each well was evaluated at the indicated time points and converted to collagen, as described in Materials and Methods. Data are the mean ± SD of the results obtained in three different experiments for each sample. (C) Second harmonic generation images of hOSTs at day 6, day 11, day 14, and 18 alone or in direct co-culture with HSCs, performed at O_2_ 20% or at O_2_ 5%. All images were acquired through a 63x (1.2 NA) objective (scale bar  = 30 µm). (D) Expression of type II and III collagen by hOSTs after 6 days of differentiation, as compared to hMSCs, evaluated by real-time RT-PCR.

In order to recapitulate the relationship between Mk and osteoblast differentiation and maturation [19], co-culture experiments were performed. Human umbilical cord blood (hUCB) derived progenitor cells were cultured on hOSTs at day 6 of differentiation, with co-cultures maintained for twelve days in the presence of growth factors specific for progenitor differentiation towards the megakaryocytic lineage. While Mks were differentiating and maturing (Figure 2A), a progressive increase in type I collagen release was observed as revealed by hydroxyproline quantification (Figure 2B). Interestingly, when co-cultures were maintained in hypoxic conditions, the concentrations observed at each time point were significantly higher than those reported for hOST cultures alone or co-cultures at normoxic conditions (Figure 2B). As expected, these results indicate that hemopoietic progenitors and Mks promote and sustain bone formation and matrix deposition. Likewise, oxygen tension also plays a crucial role in promoting type I collagen release.

We further investigated the deposition of type I collagen with second harmonic generation (SHG) imaging, a non-invasive optical method that exploits non-linear light scattering from fibrillar collagen. Using this technology, a time course analysis of the deposition of fibrillar type I collagen from hOSTs revealed that, hOSTs in co-culture with HSCs at 5% oxygen tension organized regularly oriented collagen fibers. These fibers were evident after eleven days of culture, with the presence of aligned fibers increasing progressively until day 18 of culture when fibrils were so abundant that they totally covered the underlying osteoblastic layer (Figure 2C). To exclude the possibility that SHG images were derived from the presence of other fibrillar collagen types produced by hOSTs, we analyzed the expression of type II and III collagens and these were negative at day 6 of differentiation (Figure 2D) and did not increase thereafter (data not shown). Further type II and III collagen expression was absent during the culture of osteoblasts alone and hOST-HSC cocultures at both oxygen tensions (data not shown). Therefore, since hOSTs are known to express only type I, II and III collagens, we conclude that both the SHG images and hydroxyproline assays were derived from type I collagen only.

### Influence of hMSCs and hOSTs on HSC and Mk development

**Figure 3:**
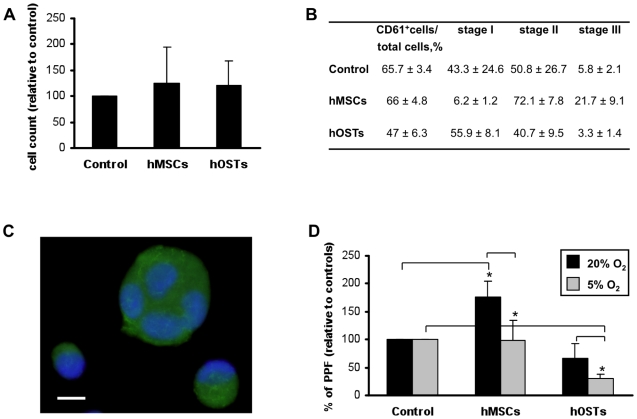
Role of the osteoblastic niche in megakaryocyte differentiation and proplatelet formation. (A) HSCs were plated in direct co-culture with hMSCs, or hOSTs or without any feeder layers (control). Adherent cells were counted as described in methods and normalized to relative control suspension cultures. (B) At day 12 of a direct co-culture cells were stained and the percentage of CD61-positive cells was determined upon conventional fluorescence microscopy analysis. Mks were assigned to distinct stages of differentiation as described in Materials and Methods. (C) Representative picture of CD61^+^ cells at different stage of maturation. (D) Proplatelet-bearing Mks were then evaluated by fluorescence microscopy, after staining with anti-alpha tubulin. Results are expressed as percentage of adherent megakaryocytes forming proplatelets and normalized to relative controls at different oxygen tensions. Data are the mean ± SD of the results obtained in three different experiments for each sample. Statistical analysis by one-way ANOVA followed by Bonferroni's *t-*test was performed for unpaired observations between hMSCs and hOSTs values. Student *t-*test was performed for paired observations to relative control. *p<0.05.

The role of the osteoblastic niche in regulating HSC proliferation, differentiation and maturation was investigated by adding HSCs to the model system. CD34^+^ cells were plated on a confluent monolayer of hMSCs or hOSTs at day 6 of differentiation as reported above, and cultured in the presence of thrombopoietin for twelve additional days. Controls were performed by plating CD34^+^ cells in suspension cultures, supplemented with the same media and growth factors. In our system when HSCs were cultured with adhesion to either hMSCs or hOSTs, cell proliferation was not affected with respect to controls (Figure 3A). These data point to the fundamental regulatory role of the physical interactions between HSCs and their microenvironment in the regulation of hemopoiesis.

Moreover the effects of different co-culture environments on Mk differentiation and maturation were examined over time. Co-cultures of HSCs with both hMSCs and hOSTs over a 12-day culture resulted in sustained Mk differentiation and no significant differences in Mk outputs (Figure 3B). However, Mk maturation stage classification, according to standard criteria [20], revealed significant differences in the maturation profiles between HSC-hMSC and HSC-hOST co-cultures (Figure 3B), indicating that hMSCs did not influence differentiation, but rather maturation of Mks. To clarify the Table in Figure 3B, we included a representative image of CD61^+^ cells at different stages of maturation from which counts were made (Figure 3C). In order to study complete maturation of Mks we analyzed proplatelet formation (PPF) and we found that Mks, differentiated in adhesion to hMSCs, extended a significantly higher number of proplatelets when compared to control (Figure 3D). Interestingly hypoxia completely suppressed this effect (Figure 3D). Further hypoxia determined a significant decrease of PPF on hOSTs demonstrating that, in this in vitro model of osteoblastic niche, cellular and physical parameters combine to inhibit HSC maturation within the osteoblastic niche (Figure 3D).

### Role of integrins alpha2beta1 and VLA-4 on megakaryopoiesis

**Figure 4:**
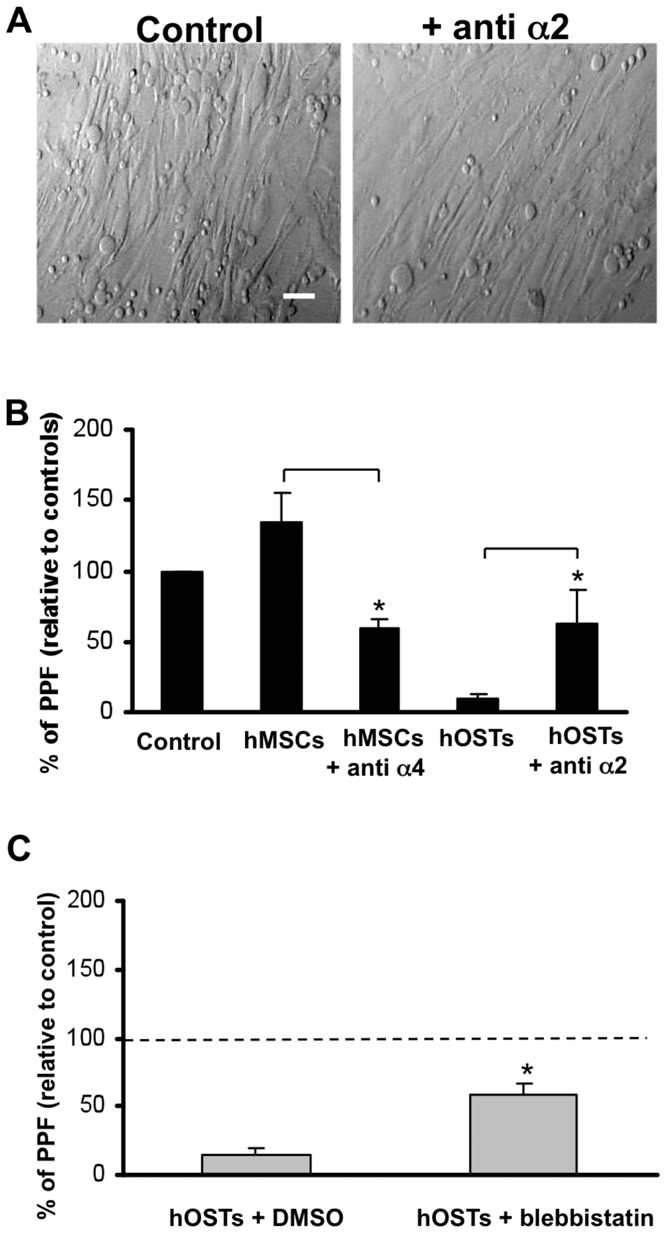
Involvement of type I collagen and VCAM-1 in proplatelet formation. Megakaryocytes, differentiated in suspension cultures for twelve days, were incubated for 30 min with unrelated isotype-matched IgG (control), the monoclonal antibody anti-integrin alpha2, clone P1E6, or anti-integrin alpha4, clone P1H4. Incubated cells, as well as control cells, were allowed to adhere to hMSCs or hOSTs or plated in suspension (control). (A) A reduction of about 30% in Mk adhesion in the presence of the anti-alpha2 antibody on hOSTs was observed, as shown in contrast phase images (scale bar  = 100 µm). (B) The percentage of adherent megakaryocytes extending proplatelets was evaluated after 16 hours. Significant difference in PPF is indicated in comparison to Mks in adhesion to hMSCs or hOSTs without any antibody pre-incubation. (C) Human megakaryocytes were treated with 100 µM blebbistatin or with DMSO for 30 min at 37°C prior to being plated onto hOSTs. PPF was evaluated upon incubation for 16 h. Results are reported as percentage proplatelet-forming cells compared with the corresponding control sample (Mks in suspension), indicated by the dotted line. Data are the mean ± SD of the results obtained in three different experiments for each sample. *p<0.05.

Since it was shown that in normoxic conditions, adhesion of Mks to endothelial cells promotes PPF through a mechanism mediated by VCAM-1 [3], we investigated the same pathway in our system. hMSCs, and not differentiated hOSTs, constitutively expressed vascular cell adhesion molecule 1 (VCAM-1) that was also revealed on the membrane surface by cytofluorimetric analysis. Interestingly, as already demonstrated by others [21], [22], in our system hypoxia also inhibited VCAM-1 expression in hMSCs, demonstrating a significant decrease in VCAM-1 expression at each time point of culture at 5% oxygen tension when compared to those performed at 20% oxygen tension (data not shown). On this basis, to further investigate the regulation of Mk differentiation and maturation by contact with hMSCs and hOSTs [23], mature Mks were plated on confluent layers of hMSCs in the presence of unrelated IgG or monoclonal antibody P1H4 anti-integrin VLA-4, or on confluent layers of hOSTs, on differentiation day 18, in the presence of unrelated IgG or monoclonal antibody P1E6 anti-integrin alpha2beta1. In each case, suspension cultures were used as controls. In terms of cell attachment, when Mks were plated on hMSCs, the presence of anti-VLA-4 did not alter adhesion, while there was a reduction of about 30% in Mk adhesion in the presence of the anti-alpha2 antibody on hOSTs (Figure 4A). Further, as shown in Figure 4B, upon incubation with VLA-4 antagonist, PPF was significantly decreased. These results indicate that alpha(4)beta(1) integrin (VLA-4) and its ligand VCAM-1 axis play a central role in promoting PPF by mature Mks co-cultured with hMSCs.

In parallel experiments we demonstrated the ability of anti-alpha2 antibody to relieve PPF inhibition by hOSTs (Figure 4B). The integrin involvement in the mechanisms described seemed to be consistent with the type of co-culture: in fact no changes were observed upon incubation of hMSC-HSC co-culture with anti-alpha2 antibody or hOST-HSC co-culture with anti-VLA-4 (data not shown).

Previous investigations of the signaling cascades associated with regulation of PPF by type I collagen via integrin alpha2beta1 revealed the involvement of the Rho/ROCK pathway [24]. As ROCK is involved in the phosphorylation of the myosin light chain, this suggests that the actin-myosin cytoskeleton, specifically the ATPase activity of myosin-IIA, plays a role in the regulation of PPF. To confirm this, co-cultures of Mks were treated with the selective antagonist of myosin IIA ATPase activity blebbistatin, in the physiological environment of 5% oxygen tension. As shown in Figure 4C, the percentage of PPF generated by blebbistatin-treated Mks in co-culture with hOSTs was significantly higher than when these cells were treated with DMSO alone. These data further confirm the essential role of integrin alpha2beta 1 in PPF inhibition within the model of the osteoblastic niche.

All together these results indicate that the osteoblastic niche represents a dynamic space where hMSCs differentiated into hOSTs physically interact with HSCs and provide a quiescent environment, while surrounding undifferentiated hMSCs might support HSC differentiation towards the megakaryocytic lineage. Furthermore, in this system hypoxia represents one of the key elements in the inhibition of HSC differentiation by promoting bone formation while inhibiting the expression of hMSC adhesion molecules.

### Morphological analysis of proplatelets

**Figure 5:**
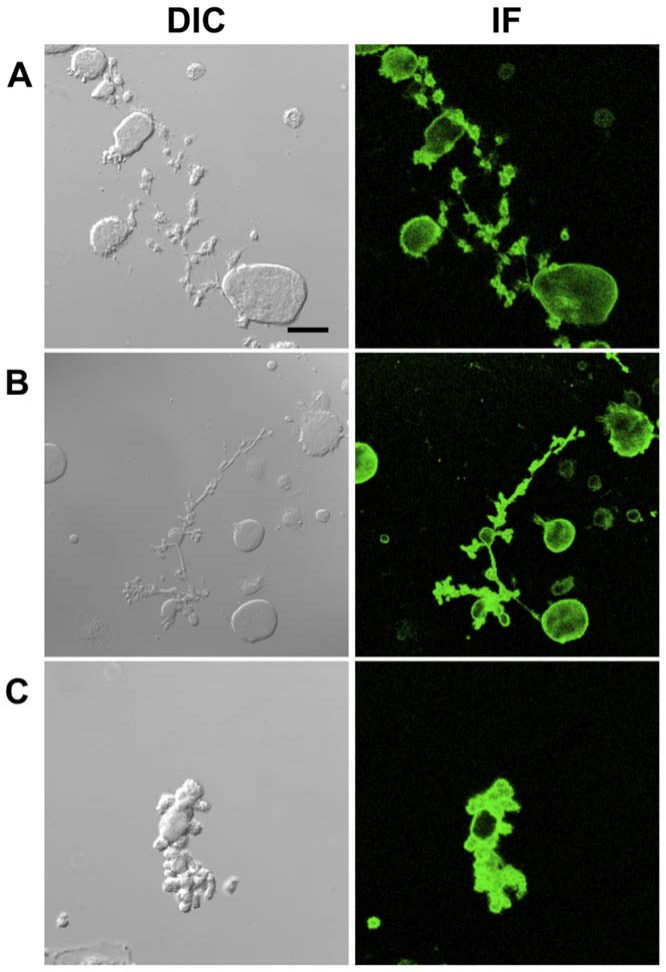
Morphological analysis of proplatelets. Differential interference contrast (DIC) and immunofluorescence (IF) images of a megakaryocyte extending proplatelets in suspension (A), in adhesion to hMSCs (B), or to hOSTs (C), as revealed by staining for CD61 (scale bar  = 40 µm).

Analysis of cell morphology by phase contrast microscopy revealed clear differences in the general architecture of the proplatelets extended by Mks adherent to hOSTs as compared to both control cultures and Mks adherent to hMSCs. Immunofluorescence analysis of CD61 staining revealed the presence of self-wrapped proplatelets [25], [26] derived from hOST co-cultures (Figure 5C) with respect to controls (Figure 5A) and hMSC co-cultures (Figure 5B) that displayed normal, long extended proplatelets. In almost 90% of the PPF from hOST co-cultures, proplatelet shafts appeared wrapped and with a reduced number of branches. In addition, proplatelet tips did not exhibit their typical round shape and swellings were not evident at the end of the proplatelet shaft as seen in controls and hMSC co-cultures [25], [26]. These results provide morphological support for the regulatory mechanisms that govern platelet maturation and release within the osteblastic niche.

## Discussion

The development of the present model to systematically study the functional interactions between HSCs and their niche, mimics and combines *in vitro* the biochemical and physical parameters of the osteoblastic niche. By including a new non-linear imaging approach based entirely on endogenous sources of optical contrast, this system allows for the non-invasive and dynamic characterization of key structural and architectural features of the model [27], [28]. To date, proplatelet formation and platelet release have been visualized by multiphoton intravital microscopy in intact bone marrow (BM) [29] and HSC homing and proliferation within the bone marrow have been traced through the development of *ex-vivo* real time imaging technology [30]. These studies have described the osteoblastic niche as a very dynamic environment where HSCs can be exposed to both endosteum and vascular signals that differently determine their fate. Despite this improvement in the knowledge of functional bone marrow niches, the mechanisms underlying the relationships between HSCs and their environment are not understood, especially in humans where invasive approaches are not possible. In this context, the ability to develop and utilize an *in vitro* model to account for the influences of bone marrow microenvironments is essential to elucidating the process involved in cell maturation, migration and function.

The present work demonstrates that HSCs differentiating in Mks and hypoxia promote not only hMSC differentiation towards the osteoblastic lineage, but also enhances fibrillar organization of type I collagen released from hOSTs, demonstrating for the first time that cellular and physical parameters are fundamental for both composition and structure of the osteoblastic niche [31], [32], [33]. Importantly, by SHG-based imaging, an increase in type I collagen fibril deposition was observed when HSCs were added to the system under hypoxic conditions, with regularly ordered fibrils filling the well by the end of co-culture. These results clearly demonstrated that the combination of lower oxygen tension and HSCs formed a niche favorable to fibrillar type I collagen deposition. Thus, this environment constituted a quiescent site for HSCs for differentiation through the megakaryocytic lineage, but not to complete maturation and extension of proplatelets, even in the presence of growth factors. These results are consistent with a model in which the coexistence of hypoxia, HSCs and Mks promotes type I collagen deposition in the osteoblastic niche and thus restrains Mks from extending proplatelets and prevents the premature release of platelets. Moreover, this model demonstrated that PPF inhibition by type I collagen depended on its structural properties, as detected by SHG-based imaging. To support these findings, recent work has shown that Mk adhesion to collagen type IV, an ECM protein located around the marrow vessels, supports PPF. Overall, the mechanisms for the differential effects of collagen subtypes on PPF are still unknown, but preliminary evidence indicates that these differences may be ascribed to the distinctive structural properties of the collagens [6], [15].

The inhibitory effect of type I collagen on PPF is mediated by the interaction with integrin alpha2beta1 [13], [34]. Using a specific antibody to block integrin alpha2beta1, Mk adhesion to hOSTs was reduced, but their ability to extend propletelets was restored, eliminating the inhibitory effect induced by the binding of released type I collagen to its adhesive receptor on the Mk membrane. Previous work found that the inhibitory action of type I collagen on PPF is dependent on myosin IIA [6], the only isoform expressed in Mks and platelets [35] and is encoded by the *MYH9* gene, whose mutations cause thrombocytopenia [36]. Two recent reports have implicated myosin IIA in the Rho/ROCK-mediated inhibition of PPF [24], [37]. The results presented here support these previous findings of myosin-IIA as a key element in Mk regulation, demonstrating that inhibition of myosin IIA counteracts the ability of type I collagen to repress PPF in a physiological setting of the osteoblastic niche. Together, these results indicate that type I collagen can negatively control PPF as long as the functionality of myosin is preserved. Interestingly, previous work in the lab showed that megakaryocytes obtained from the peripheral blood of patients with specific *MYH9* mutations present a loss of function of myosin-IIA and completely lose the physiologic suppression of PPF exerted by type I collagen [26]. Finally, as it is known that hOSTs produce several cytokines that are critical for hemopoiesis and megakaryopoiesis, this work extends these observations by demonstrating that physical contact between the osteoblastic niche environment and Mks can inhibit PPF that is otherwise enhanced when interactions between HSCs and their environment are missing [38]. These results strongly support the conclusion that different elements combine to regulate Mk maturation within the osteoblastic niche and a perturbation of a single element can cause premature proplatelet extension and ineffective platelet release into the blood stream.

Different cells, other than hOSTs, derive from hMSCs into the bone marrow and support megakaryopoiesis [38]. Moreover, it has been shown that hMSCs stimulate Mk and platelet production from HSCs [39]. Thus, opposite signals are given to HSCs by hMSCs and differentiated hOSTs. This work demonstrated that hMSCs, and not hOSTs, constitutively express VCAM-1 [40], and contribute to Mk development and PPF [41] by HSCs in co-culture with hMSCs. In addition, hypoxia significantly down regulated VCAM-1 expression by hMSCs resulting in a more limited effect on PPF that was comparable to the relative control. These results could be explained by the existence of a possible mechanism for inhibition of PPF in the less oxygenated areas that are typical of the quiescent osteoblastic niche. By using a specific antibody against integrin alpha4, the involvement of the VCAM-1/VLA-4 axis in this regulatory mechanism was revealed, as blocking of alpha4 suppressed the effect of hMSCs on PPF.

In conclusion, this study provides important new elements in the understanding of the regulatory pathways for Mk maturation and PPF within the bone marrow space. In particular, the development of an in vitro model that combines the cellular, physical and biochemical features of the osteoblastic niche to understand the physiological mechanisms that regulate of megakaryopoiesis. In future work, diseased cells may be incorporated into the model, providing insight into the pathophysiology of defects of platelet production as well as the ability to test the targets of new therapies on normal and defective megakaryopoiesis [26], [42].

## Materials and Methods

### Materials

Human mesenchymal stem cells (hMSCs) were from Clonetic-Poietics (Walkersville, MD). Dulbecco's modified Eagle's medium (DMEM), fetal bovine serum (FBS), penicillin-streptomycin, non-essential amino acids and basic fibroblast growth factor (bFGF) were from Gibco (Carlsbad, CA). Bovine purified type I collagen was provided by Prof. Tira and Dr. Gruppi (University of Pavia). Alizarin red solution (2%), blebbistatin, mouse anti-human type I collagen and Poli-L-Lysine solution was from Sigma (St. Louis, MO). Human umbilical cord blood was from The National Disease Research Interchange (Philadelphia, PA). Lympholyte was from Accurate Chemical and Scientific Corporation (New York, NY). Immunomagnetic separation system was from Miltenyi Biotech (Auburn, CA). Stem Span medium was from Stem-Cell Technologies (Vancouver, Canada). Recombinant human TPO, interleukin (IL)-6 and IL-11 were from PeproTech (Rocky Hill, NJ). The following antibodies have been used: mouse monoclonal anti-CD61, clone SZ21, from Immunotech (Marseille, France); mouse anti-human integrin alpha2, clone P1E6, and anti-human integrin alpha4, clone P1H4, from Chemicon International (Billerica, MA), PE-conjugated mouse anti-human VCAM1 from BD Pharmingen (San Diego, CA). Alexa Fluor 488-conjugated chicken anti-mouse, Mowiol 4–88 and Hoechst 33258 were from Molecular Probes (Eugene, OR). Oligonucleotide primers were from Applied Biosystems (Foster City, CA). WesternBreeze Chromogenic Western Blot Immunodetection Kit was from Invitrogen (Carlsbad, CA).

### hMSCs and Osteoblast culture

Human mesenchymal stem cells (hMSCs) were harvested and cultured according to previously described protocols [41]. Briefly, hMSCs were cultured in Dulbecco's modified Eagle's medium (DMEM), supplemented with 10% fetal bovine serum, 100 U/mL penicillin-streptomicin, 100 U/mL non-essential amminoacids, and 1 ng/mL basic fibroblast growth factor (bFGF) at 37°C in a 5% CO_2_ fully-humidified atmosphere. To induce osteoblast differentiation, hMSCs were cultured in Dulbecco's modified Eagle's medium, supplemented with 10% fetal bovine serum, 100 U/mL penicillin-streptomicin, 100 U/mL non-essential amminoacids, 100 nM Dexamethasone, 10 nM β-Glycerol phosphate and 0.05 mM Ascorbic acid at 37°C in a 5% CO_2_ fully-humidified atmosphere, either at 5% O_2_ or 20% O_2_. Cell culture medium was replenished every 2–3 days.

### Total RNA extraction and real-time RT–PCR analysis

hMSCs and hOSTs were collected at different days of culture and reverse transcription-polymerase chain reaction (RT-PCR) analysis was performed. Total RNA was isolated using the RNeasy mini kit (Qiagen, Valencia, CA) and reverse transcribed to cDNA using SuperScript II reverse transcriptase (Invitrogen, Carlsbad, CA) by following manufacturer's instructions. For reverse transcription, 5 µl of RNA sample with 45 µl of RNase-free water was used to synthesize cDNA using a High-Capacity cDNA Archive Kit (Applied Biosystem, Foster City, CA). Oligonucleotide primers for type I collagen (Col I) and Alkaline Phosphatase (ALP) and VCAM-1 transcripts were used in distinct PCR reactions together with primers specific for the GAPDH gene, which was selected as the housekeeping gene for determining relative expression levels. All gene assays were pre-made and characterized by Applied Biosystems following standard criteria. When bone formation gene expression by hOSTs were analyzed, negative controls were represented by hMSCs. In VCAM-1 RT-PCR experiments hMSCs grown at 20% oxygen tension were considered as standard reference. The PCR amplifications were performed using ampli-Taq (Applied Biosystems, Foster City, CA) and a programmable thermal controller Mx3000P QPCR system (Stratagene, La Jolla, CA).

### Dot-blot for type I collagen

hMSCs and hoSTS were washed with ice-cold PBS before proteins were extracted in RIPA buffer (20 mMTris–HCl pH 7.4, 137 mM NaCl, 10% (v/v) glycerol, 1% (v/v) Triton X-100, 2 mM EDTA) containing 0.5 mM protease inhibitor, phenylmethylsulphonylflouride (PMSF). After 30 min in ice, extracts were centrifuged for 10 min at 10,000×g, 4°C, and the protein content of the supernatants was determined by Bradford assay (Biorad). 10 µg of each sample including 40 ng of a positive control (purified type I collagen) were applied directly to nitrocellulose membranes. Membranes were blocked with 6% bovine serum albumin (BSA) in Tris-buffered saline containing 0.05% Tween 20 at room temperature for 1 h and then incubated overnight at 4°C with a primary monoclonal antibody against human Type I collagen diluited 1∶1000 and an alkaline phosphatase-conjugated anti-mouse secondary antibody solution (WesternBreeze Chromogenic Western Blot Immunodetection kit). The blots were developed and detected using the same Chromogenic detection kit.

### Calcium deposition assay

Calcium deposition was revealed by alizarin red assay at day 6, 11 and 18 of differentiation. Alizarin red solution (2%) was prepared fresh and adjusted to pH 4.1–4.3. The cells were washed with PBS, fixed with PFA 3% 15 min and deionized water before 2% alizarin red solution was added. The solution was removed after 10 min and the cells were rinsed with deionized water. Conventional microscopy was performed using a 10x objective through an Axiovert S100 microscope with a Sony Exwave HAD 3CCD color video camera and the ImageJ acquisition software (NIH).

### Total collagen quantification

To assess the amount of collagen produced by the cells, the amount of hydroxyproline was measured using a previously described method [44]. The chloramine T solution and aldehyde-perchloric acid solution used for the assay were prepared as follows: Chloramine T (sodium *N*-chloro-*p*-toluenesulfonamide) 0.3535 g was dissolved in 5.175 mL of water, and then 6.5 mL of n-propanol and 13.325 mL of pH 6 buffer (citric acid monohydrate 25 g, acetic acid 6 mL, sodium acetate trihydrate 60 g, sodium hydroxide 17 g to 500 mL) were added. Aldehyde-perchloric acid solution was freshly made: 3.75 g of *p*-dimethylaminobenzaldehyde was dissolved in 15 mL of n-propanol, and 6.5 mL of perchloric acid was added slowly. First, the cells and matrices were recovered from each well and hydrolyzed with 6 N HCl at 115°C for 18 h on a heating block. The hydrolysate was then transfered to eppendorf tubes and placed on a heating block at 100°C overnight to allow acid to evaporate. The resultant hydrolysate was dissolved in 3 mL water and evaporated at 95°C. Finally, it was resuspended in 1.5 mL water and used as a sample. A volume (250 µL) of chloramine T solution was added to 500 µL of the sample and the mixture left for 20 min at room temperature. Finally, 250 µL of p-dimethylaminobenzaldehyde was added and the mixture incubated for 15 min at 60°C. After cooling, the absorbance was read at 550 nm. Total hydroxyproline concentration was then multiplied by a factor of 7.46 to give the total collagen concentration as described in literature [45].

### Second harmonic generation microscopy for analysis of released type I collagen by osteoblasts

Second harmonic generation (SHG) micrographs were acquired on a Leica DMIRE2 microscope with a TCS SP2 scanner (Wetzlar, Germany). The excitation light source was a Mai Tai tunable (710–920 nm) titanium sapphire laser emitting 100 fs pulses at 80 MHz (Spectra Physics, Mountain View, CA). Osteoblasts were placed onto 12 mm coverslips previously coated with poli-L and excited at 800 nm. SHG images were acquired in a transmission geometry through a filter centered at 400 nm (Chroma hq400/20m-2p) with a 20-nm bandpass. Analysis was performed with the Leica Confocal Software (Wetzlar, Germany) and Image J (NIH, Bethesda, MD).

### Flow cytometry analysis

hMSCs were collected using Trypsin 0.05% and washed with PBS containing 50 mM EDTA and 0.5% fetal bovine serum and then incubated with a PE-conjugated anti-VCAM1 antibody at 4°C for 30 minutes. After being washed twice with PBS, flow cytometric analysis was performed. VCAM1 expression was quantified using a FACS®Calibur flow cytometer (Becton Dickinson), with 50,000 events recorded for each sample. Experiments were repeated at least twice under the same conditions and settings.

### Co-cultures experiments

Co-cultures with direct cell–cell contact were performed by plating 7×10^4^ human umbilical cord blood CD34^+^ cells directly onto confluent layers of hMSCs or hOSTs at day 6 of differentiation, in 24 well plates. Co-cultures were maintained for up to 12 days as described below in the Mk differentiation method. Controls were performed by seeding CD34^+^ cells alone in suspension cultures. Cultures were performed at either 5% O_2_ or 20% O_2_ for the entire experimental period. During co-culture the amount of collagen released by hOSTs and its orientation through second harmonic generation images were analyzed and compared to hOSTs cultured alone at the same condition of co-cultures. Mk and proplatelet formation were monitored as described below.

### Differentiation of megakaryocytes from cord blood and PPF evaluation

CD34^+^ cells from human cord blood were separated as previously described [46] and cultured in different co-culture conditions for 12 days in Stem Span medium supplemented with 10 ng/mL TPO, IL-6, IL-11 at 37°C in a 5% CO_2_ fully-humidified atmosphere. Medium was changed at day 3, 7 and 10 of co-culture. At the end of co-culture, cell count to analyze the output of HSCs in suspension and in adhesion to different cell types was performed by phase-contrast microscopy. Briefly: 20 fields were taken randomly by phase-contrast microscopy and the larger cells counted. To evaluate Mk yield a small sample of suspension culture was cytospun onto glass coverslips, while a hOST-HSC and a hMSC-HSC co-culture wells were sacrificed upon staining with anti-CD61 antibody. Mks were identified on the basis of CD61 expression, and assigned to distinct stages of maturation according to the standard morphological criteria [23]. Evaluation of Mks by phase contrast and immunofluorescent microscopy resulted in comparable results.

The percentage of Mks extending proplatelets was evaluated after 12 days of co-culture. Proplatelet-bearing Mks, in adhesion to feeder layers in the direct co-culture or in suspension in the indirect co-culture, were counted by differential interference contrast (DIC) microscopy using a Leica DMIRE2 microscope with a TCS SP2 scanner (Wetzlar, Germany) through a 63x objective (1.2 NA). Afterwards, Mks extending proplatelet in the indirect co-culture cultured were harvested, or previously gently removed from the feeder layers when cultured in a direct cell-cell contact, and cytospun onto glass coverslips before being stained with the antibody against CD61 (see below). Mks forming proplatelets were identified as large CD61^+^ cells extending long filamentous structures. The extent of PPF was calculated as the percentage of proplatelet-bearing CD61^+^ cells with respect to total CD61^+^ cells. Evaluation of PPF by phase contrast and immunofluorescent microscopy resulted in perfectly comparable results.

In some experiments, CD34^+^ cells were cultured in suspension for 12 days and 1×10^5^ mature Mks were then pre-incubated for 30 minutes with 30 µg/mL mouse anti-human integrin alpha2 or anti-human integrin alpha4, prior to being plated. Negative controls were prepared with cells incubated with identical concentrations of unrelated isotype-matched IgG.

### Immunofluorescence analysis

Mks were cytospun onto glass coverslips and fixed in 3% paraformaldehyde for 20 minutes at RT. Upon washing with PBS, cells were blocked with 3% BSA in PBS for 1 hour at RT. Cells were then incubated for 1 hour at RT with the primary monoclonal antibody mouse anti-CD61, clone SZ21, 1∶100, diluted in PBS. After washing with PBS, cells were incubated with 10 µg/mL of the secondary antibody conjugated with Alexa Fluor 488 in PBS for 1 hour at RT. Specimens were mounted in Mowiol 4–88. Images were acquired using a Leica DMIRE2 microscope with a TCS SP2 scanner (Wetzlar, Germany) through a 63x objective (1.2 NA). For each specimen, at least 100 Mks were evaluated.

### Statistics

Values were expressed as mean ± SD (standard deviation). Analysis by one-way ANOVA was followed by *post-hoc* testing (Bonferroni's *t-*test). Student *t-*test was performed for paired observations. A value of *p*<0.05 was considered statistically significant. Statistical analysis was carried out using SigmaStat 3.0 software. All experiments were independently replicated at least three times.
